# Phosphazene-Based Porous Polymer as Electrode Material for Electrochemical Applications

**DOI:** 10.3390/polym18030366

**Published:** 2026-01-29

**Authors:** Ekaterina A. Karpova, Alexander A. Sysoev, Ilya D. Tsvetkov, Alexey L. Klyuev, Oleg A. Raitman, Mikhail A. Soldatov

**Affiliations:** 1 Department of Chemical Technology of Polymeric Composite Paints and Coatings, Mendeleev University of Chemical Technology, Miusskaya Sq. 9, Moscow 125047, Russia; ekaterinaakarpova@yandex.ru (E.A.K.); shura.sys@yandex.ru (A.A.S.); tsvetkov.i.d@muctr.ru (I.D.T.); klyuevchem@mail.ru (A.L.K.); raitman.o.a@muctr.ru (O.A.R.); 2 Laboratory of Structural and Morphological Research, A.N. Frumkin Institute of Physical Chemistry and Electrochemistry of Russian Academy of Sciences, Leninskiy Pr. 31-4, Moscow 119071, Russia

**Keywords:** phosphazene, porous polymers, cross-linking, carbon materials, electrode materials

## Abstract

Porous highly cross-linked polymer (PIP) was synthesized by a polycondensation reaction between hexachlorocyclotriphosphazene and piperazine. The obtained polymer has a surface area of 76.9 m^2^/g and a mesoporous structure. After carbonization, the obtained product (PIP-C) has a surface area of 177 m^2^/g. The obtained carbon product contained nitrogen and phosphorus heteroatoms, which leads to a higher specific capacitance (155.6 F/g) and catalytical activity in the electroreduction of oxygen (15.9 A/g). This work shows the possibility of the use of such porous phosphazene polymers as precursors for heteroatom-doped carbon materials, which might be used in electrochemical devices like electrodes for supercapacitors or metal-free electrocatalysts in fuel cells.

## 1. Introduction

Currently, various carbon materials like activated carbons, graphite, or pyrolytic carbon can be used as electrode materials for modern power sources such as Li-ion batteries, supercapacitors, fuel cells, etc. [1,2,3]. These materials possess advantages such as high surface area, porosity, good electrical conductivity, and light weight [4,5]. However, they also suffer from shortcomings such as capacity losses when voltage sweep rate increases, low wettability at high concentrations of polymer, high degradability after long usage, etc. [6,7,8]. One of the popular approaches to solve these problems involves doping of the carbon materials with heteroatoms [1,9]. In general, the introduction of heteroatoms such as phosphorus and nitrogen leads to an increase in functional centers, the formation of additional defects in material, changing of charge density, and expansion of operating voltages [10,11,12,13,14,15]. Phosphorus may therefore lead to an increase in graphitization and surface area. It also decreases undesirable oxidative processes, which in turn improves the electro-chemical properties of the carbon electrode material [16,17,18]. On the other hand, nitrogen atoms improve wettability with electrolyte solution, which in turn leads to an increase in pseudocapacitance as the electrolyte ions may interact with active nitrogen atoms of the carbon electrode [19,20,21]. The currently known methods for doping usually have low efficiencies due to their technological complexity, the use of hazardous reactants, and high cost [22,23,24]. The use of highly cross-linked porous polymers based on cyclophosphazenes might be a promising approach for the preparation of electrode materials doped with phosphorus and nitrogen heteroatoms [25,26,27,28,29,30,31,32]. In [33], a heteroatom-doped carbon material was prepared from hexachlorocyclotriphosphazene (HCCP) and DOPO-HQ. The obtained material possessed a high surface area, a microporous structure, and good electrochemical properties. In [34], doped porous carbon was prepared by carbonization of cross-linked polyphenoxyphosphazene. This material exhibited quite high specific capacitance in two-electrode and three-electrode cells. All these works show high perspectives in the use of phosphazene polymers as precursors for heteroatom-dope carbon materials with improved electrochemical activity. Here, we have synthesized a phosphazene-based porous polymer through polycondensation of HCCP with piperazine, which was subsequently carbonized to obtain a heteroatom-doped carbon material with high specific capacitance and catalytical activity in electrochemical reactions. Despite piperazine-phosphazene polymers being synthesized previously, we are not aware of any works where such polymers were studied in terms of porosity and where these polymers were used as precursors for the preparation of carbon materials. These experiments also show the possibility of the use of this material as a metal-free (especially Pt-free) electrocatalyst for low-temperature oxygen reduction in fuel cells [35].

## 2. Materials and Methods

### 2.1. Materials

Unless otherwise noted, all the used chemicals were purchased from commercial suppliers and were used as received. Piperazine (99%), triethylamine (99%), and hexachlorocyclotriphosphazene (98%) were purchased from Macklin (Shanghai, China). Tetrahydrofuran (THF) was dried with sodium and benzophenone and was freshly distilled before use.

### 2.2. Synthesis of PIP

PIP was synthesized according to the previously reported procedure [36]: 1.485 g (17.25 mmol) of piperazine and 2.5 mL (5.75 mmol) of triethylamine (TEA) were dissolved in 60 mL of THF in three-necked flask, equipped with reflux condenser and magnetic stirrer. Then, 2 g (5.75 mmol) of HCCP dissolved in 60 mL of THF was added dropwise. Then, the temperature was raised to 60 °C and the reaction mixture was stirred for 72 h in an argon atmosphere. The solid polymer was filtered, washed, and extracted with chloroform for 5 h and dried under vacuum at 50 °C for 6 h. PIP was obtained as a white powder with a yield of 3.34 g.

### 2.3. Preparation of Carbonization Product PIP-C

Thermal treatment of PIP was carried out in a tube furnace under nitrogen flow. The rate of nitrogen flow was 50–60 cm^3^/s. The heating mode was performed according to Table 1.

### 2.4. Electrode Preparation

The PIP-C grinding was carried out for 1 h at 800 rpm in an agate mortar with agate balls on a Pulverisette 7 Ball mill from Fritsch (Fellbach, Germany).

Catalytic ink was used to prepare a thin layer of material deposited on a disk electrode. To prepare the ink, 2 mg of the sample was weighed in an Eppendorf tube, 300 µL of water was added and subjected to ultrasonic dispersion for 30 min. A solution of Nafion suspension was prepared in a separate Eppendorf tube: 1 mL of 10% Nafion suspension was added to 200 mL of water and subjected to ultrasonic dispersion for 5 min. The Nafion suspension solution was then transferred to a sample suspension and subjected to ultrasonic dispersion for 30 min. A micropipette of 3.15 µL of the mixture was taken from the resulting suspension and applied to a surface (0.126 cm^2^) of the rotating disk electrode (RDE), which corresponds to 100 µg/cm^2^ (12.6 µg of carbon material on the entire electrode). The rotating disk electrode is made of isotropic pyrolytic graphite with a surface area of 0.126 cm^2^ pressed into Teflon.

## 3. Results and Discussion

**Figure 1:**
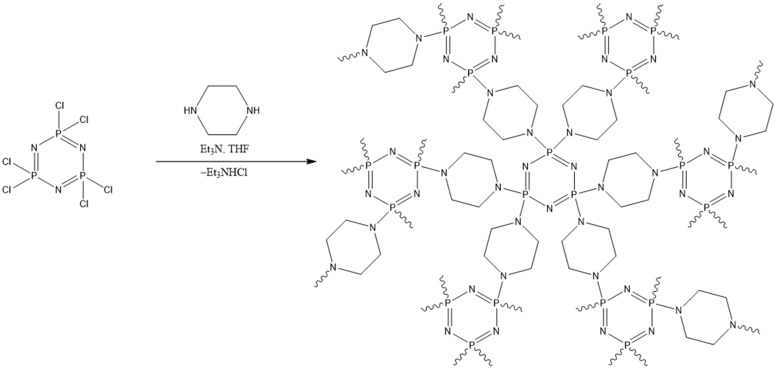
Synthetic scheme of PIP.

The phosphazene-based porous polymer (PIP) was synthesized by a polycondensation reaction between HCP and piperazin in THF solution (Figure 1). Previously, we have shown that such reaction conditions in combination with Soxhlet extraction can lead to highly porous polymer formation [36]. Here, TEA was used as an acceptor of HCl. The choice of the monomers was explained by their cyclic rigid structure, which might provide porosity to the final polymer.

**Figure 2:**
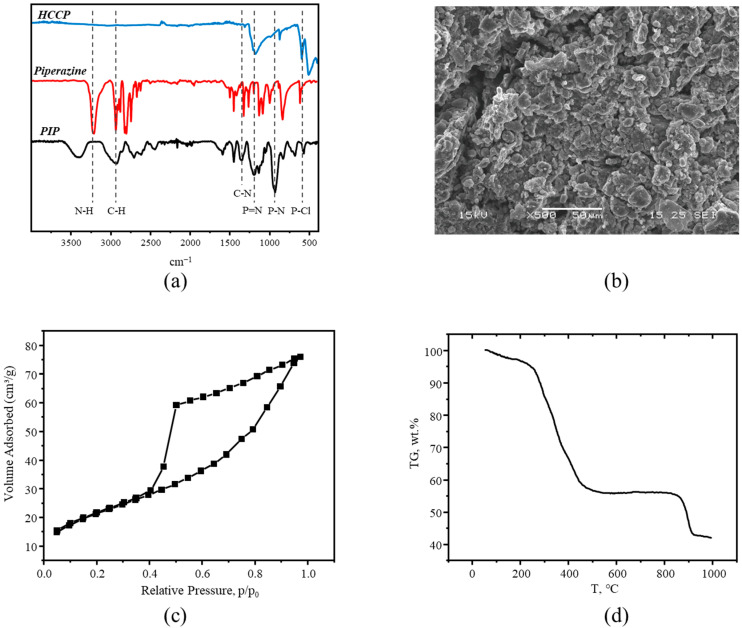
(**a**) FTIR spectra of initial monomers and PIP; (**b**) SEM image of PIP; (**c**) N_2_ adsorption–desorption isotherm of PIP; (**d**) TGA curve of PIP.

FTIR spectra of the obtained polymer are given in Figure 2a. On the spectrum of PIP, one can see that the signals at 3220 and 510 cm^−1^, corresponding to stretching vibrations of N-H and P-Cl bonds respectively, are almost absent. Also, one can observe peaks at 1180, 1380, and 2920 cm^−1^, corresponding to P=N, C-N, and C-H bonds, respectively [37,38,39]. Peaks at 2710 cm^−1^ might correspond to a side product of the reaction (triethylammonium hydrochloride).

SEM analysis (Figure 2b and Figure S1) shows that the synthesized polymer consists of flake-like particles with a size of about 5 μm. Powder X-ray analysis (Figure S2) shows that PIP has an amorphous structure in comparison with initial monomers.

The porosity and pore size distribution of the PIP were determined by low-temperature nitrogen adsorption (Figure 2c, Figures S3 and S4). As one can see, the adsorption–desorption isotherm curve can be defined as IV type according to IUPAC nomenclature, with a clear hysteresis loop, which indicates a mesoporous character of the polymer with a mean pore size of mesopores of about 3.9 nm. The surface area calculated by the BET method was 76.9 m^2^/g. Total pore volume was 0.11 cm^3^/g and micropore volume calculated by the t-plot method was 0.003 cm^3^/g.

**Figure 3:**
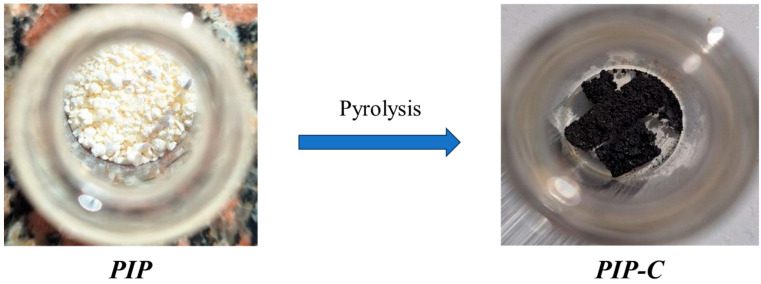
View of initial PIP and carbonized product PIP-C.

The TGA curve shows that the polymer decomposes in two steps (Figure 2d). On the first stem it starts to decompose at a temperature of 240 °C with the formation of a carbon structure doped with nitrogen and phosphorus atoms. On the second step, it decomposes at temperatures higher than 800 °C which might be due to side reactions of the carbonized product with nitrogen gas used in the analysis and other oxidation processes. From the TGA analysis the following carbonization mode was chosen: (1) fast heating till 140 °C; (2) heating from 140 to 520 °C with rate of 1 °C·min^−1^; (3) heating from 520 to 800 °C with a rate of 2.5·°C min^−1^. After that, the product was cooled naturally until reaching room temperature. The carbonized product PIP-C was obtained as a black powder (Figure 3) with the use of tube furnace under nitrogen flow with a yield of 30 wt.% relative to the initial porous polymer.

**Figure 4:**
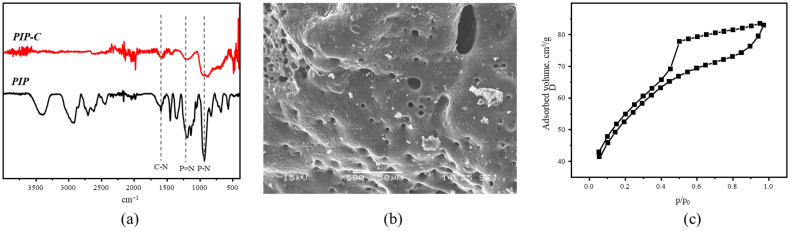
(**a**) FTIR spectra of initial PIP and PIP-C; (**b**) SEM image of PIP-C; (**c**) N_2_ adsorption–desorption isotherm of PIP-C.

From the FTIR spectrum (Figure 4a) one can see that the obtained carbon product contains C-N and P-N bonds, which are also present in the initial porous polymer (1380 and 1180 cm^−1,^ respectively). Also, one can see the full disappearance of the signal of C-H and N-H bonds (2920 and 3220 cm^−1^) which indicates successful pyrolysis. SEM images show that the PIP-C has a sponge-like morphology (Figure 4b and Figure S1). XRD analysis (Figure S2) shows that the PIP-C has two broad peaks at ~23° and ~45°, which correspond to (002) and (100) planes of carbon materials [40].

Low-temperature nitrogen adsorption shows that the PIP-C possesses higher porosity in comparison with initial PIP due to the formation of pyrolysis gaseous products. The adsorption–desorption isotherm curve (Figure 4c) can also be defined as IV type according to IUPAC nomenclature, as well as the initial PIP polymer. The surface area calculated by the BET method was 177 m^2^/g. Total pore volume was 0.129 cm^3^/g and micropore volume calculated by the t-plot method was 0.07 cm^3^/g. Based on these obtained data of pore size distribution (Figures S3 and S4), PIP-C can be defined as a mesoporous material with a mean size of mesopores of about 3.8 nm and a higher content of micropores in comparison with the initial PIP precursor.

**Figure 5:**
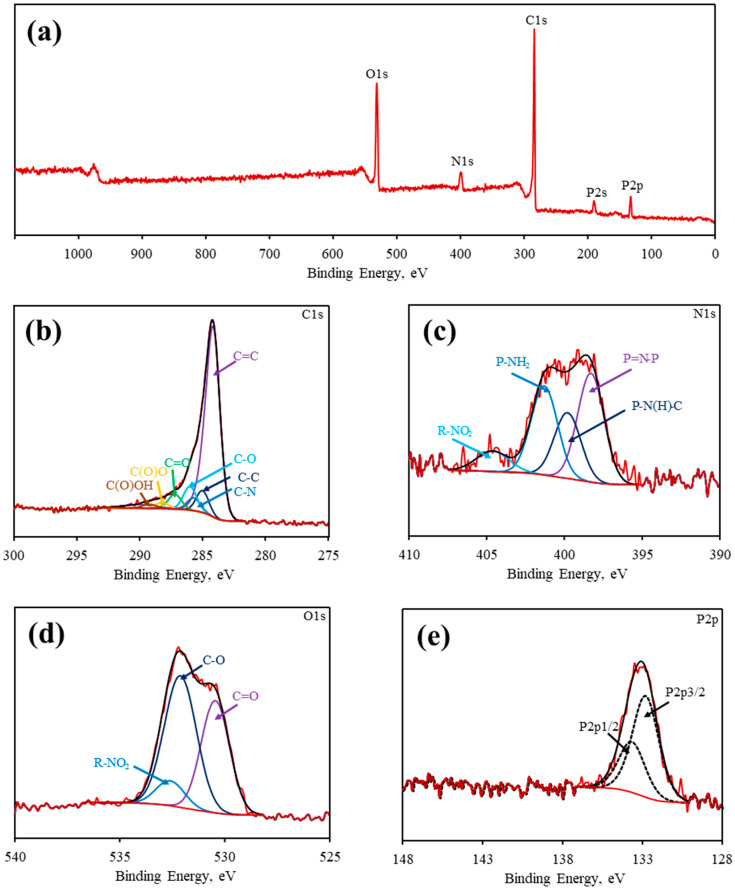
Wide scan XPS spectrum of PIP-C (**a**) and spectra of C1s (**b**), N1s (**c**), O1s (**d**), and P2p (**e**). Red lines—initial spectra, black lines—spectra after smoothing.

XPS was carried out for a deeper study of the chemical structure of the carbon material. Wide scan XPS spectrum exhibits clear peaks at 133, 284, 399, and 532 eV, corresponding to P2p, C1s, N1s, and O1s atoms, respectively (Figure 5a), indicating that all heteroatoms were successfully incorporated into the structure of carbon material. Content of N and P was 7.5 and 7%, respectively (Figure S1). Chemical states of P2p, C1s, N1s, and O1s were studied as well (Figure 5b–e). The XPS spectrum of C1s (Figure 5b) can be deconvoluted into five main peaks at 284.2, 285.0, 285.7, 286.0, and 287.2 eV, corresponding to C=C, C-C, C-N, C-O, and C=O units, respectively [24,40]. Deconvolution of XPS spectrum of N1s (Figure 5c) exhibits four main peaks at 398.3, 399.8, 401.3, and 404.6 eV corresponding to P=N-P, P-N(H)-C, P-NH_2_, and R-NO_2_, respectively. The XPS spectrum of O1s (Figure 5d) confirms the presence of previously mentioned units, while the P2p spectrum (Figure 5e) shows that phosphorus atoms are mostly in a phosphazene ring state [33]. Here, one can see that PIP-C mostly contains fragments of C=C, C=O, and P-N=P. We suppose that the presence of oxygen might be due to partial hydrolysis of residual P-Cl bonds, leading to the formation of P-OH and P=O units in the initial cross-linked polymer. These units in turn might oxidize organic substituents during pyrolysis.

**Figure 6:**
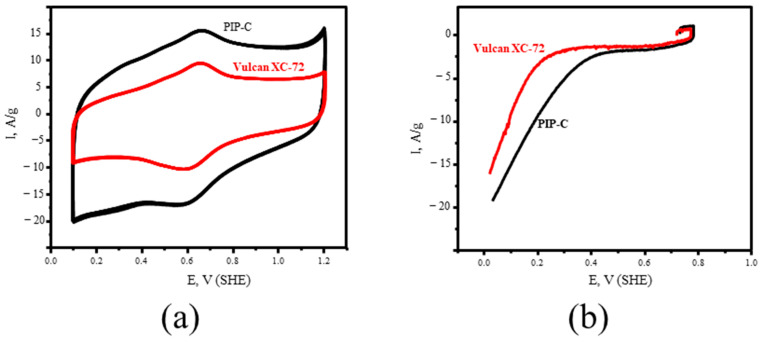
CVA (**a**) and polarization (**b**) curves of PIP-C and Vulcan XC-72.

In the next step, the PIP-C was studied in terms of its specific capacitance and electrochemical activity in a reaction of oxygen electroreduction. A commercially available carbon material widely used for electrochemical devices with the trademark Vulcan XC-72 was used for comparison of electrochemical properties. Cyclic voltammetry (CVA) curves for the studied samples in 0.5 M H_2_SO_4_ in an argon atmosphere are shown in Figure 6a. Here, one can see that PIP-C has higher values of charging current, i.e., higher double layer capacitance in comparison with Vulcan XC-72 (Table 2). Also, PIP-C’s measured specific capacitance (155 F/g) is close to that obtained for other carbon material types for supercapacitors (200–250 F/g) [41,42]. To achieve higher values of capacitance, optimal conditions are needed for electrolyte, binder/carbon ratio, etc., which is planned for future works. According to polarization curves of oxygen electroreduction (Figure 6b), PIP-C also exhibits higher catalytical activity.

Moreover, it should be noted that PIP-C, according to CVA and polarization curves, possesses a more electrochemically active surface despite the lower real surface area in comparison with Vulcan XC-72. The higher electrochemical properties can be explained by the presence of nitrogen and phosphorus heteroatoms.

## 4. Conclusions

In summary, a novel heteroatom-doped carbon material was prepared by the carbonization of a phosphazene-based porous polymer. Due to the highly rigid structure of the initial monomers, the PIP possesses porosity, as does the final PIP-C carbonization product. As the PIP-C material contains N and P heteroatoms in its structure, it exhibits good electrochemical properties in the oxygen reduction reaction and shows higher electrochemical activity in the double-layer applications (like supercapacitors) in comparison with commercial carbon material. This work opens perspectives in the simple preparation of porous carbon materials doped with phosphorus and nitrogen heteroatoms, which in turn might be potentially used as metal-free electrode materials for chemical power sources (fuel cells, metal-ion batteries, etc.) as well. In future, we plan to study various factors in more depth, like chemical structure and reaction conditions, which might influence the electrochemical properties of the final carbon materials. We also plan to find optimal conditions for the developed materials to achieve higher values of specific capacitance.

## Figures and Tables

**Table 1:** Heating mode for thermal treatment of PIP.

Temperature Interval, °C	Heating Rate, °C/min
from RT to 140	rapid heating
from 140 to 520	1.0
from 520 to 800	2.5

**Table 2:** Electrochemical properties of PIP-C and Vulcan XC-72.

Carbon Material	S_BET_, m^2^/g	Specific Capacitance, F/g	Catalytical Activity, A/g
PIP-C	177	155.6	15.9
Vulcan VX-72	230 *	58.7	12.7

* According to the technical data sheet for Vulcan XC-72.

## Data Availability

The original contributions presented in this study are included in the article and Supplementary Materials. Further inquiries can be directed to the corresponding author.

## Supplementary Materials

The following supporting information can be downloaded at: https://www.mdpi.com/article/10.3390/polym18030366/s1, Table S1: Element content on surface of PIP-C obtained from XPS data. Figure S1: SEM images of PIP (a–c) and PIP-C (d–f). Figure S2: XRD spectra of initial monomers and obtained products. Figure S3: Pore size distributions for PIP (a) and PIP-C (b) calculated by using BJH method. Figure S4: Pore size distributions for PIP (a) and PIP-C (b) calculated by using DFT method [43,44].

## References

[1] Yang F., Zhang S., Yang Y., Liu W., Qiu M., Abbas Y., Wu Z., Wu D. (2019). Heteroatoms Doped Carbons Derived from Crosslinked Polyphosphazenes for Supercapacitor Electrodes. Electrochim. Acta.

[2] Song P., Shen X., He X., Feng K., Kong L., Ji Z., Zhai L., Zhu G., Zhang D. (2019). Cellulose-Derived Nitrogen-Doped Hierarchically Porous Carbon for High-Performance Supercapacitors. Cellulose.

[3] Rawal S., Kumar Y., Mandal U.K., Kumar A., Tanwar R., Joshi B. (2021). Synthesis and Electrochemical Study of Phosphorus-Doped Porous Carbon for Supercapacitor Applications. SN Appl. Sci..

[4] Sing K.S.W. (1982). Reporting Physisorption Data for Gas/Solid Systems with Special Reference to the Determination of Surface Area and Porosity (Provisional). Pure Appl. Chem..

[5] Je S.H., Kim H.J., Kim J., Choi J.W., Coskun A. (2017). Perfluoroaryl-Elemental Sulfur SNAr Chemistry in Covalent Triazine Frameworks with High Sulfur Contents for Lithium–Sulfur Batteries. Adv. Funct. Mater..

[6] Ghazi Z.A., Zhu L., Wang H., Naeem A., Khattak A.M., Liang B., Khan N.A., Wei Z., Li L., Tang Z. (2016). Efficient Polysulfide Chemisorption in Covalent Organic Frameworks for High-Performance Lithium-Sulfur Batteries. Adv. Energy Mater..

[7] Song Z., Liang Y., Fan M., Zhou F., Liu W. (2014). Lithium-Based Ionic Liquids Functionalized by Sym-Triazine and Cyclotriphosphazene as High Temperature Lubricants. Tribol. Int..

[8] Xue R., Zheng Y.-P., Qian D.-Q., Xu D.-Y., Liu Y.-S., Huang S.-L., Yang G.-Y. (2022). A 2-D Microporous Covalent Organic Framework for High-Performance Supercapacitor Electrode. Mater. Lett..

[9] Allcock H.R., Kwon S. (1989). An Ionically Crosslinkable Polyphosphazene: Poly[Bis(Carboxylatophenoxy)Phosphazene] and Its Hydrogels and Membranes. Macromolecules.

[10] Fei S.-T., Allcock H.R. (2009). Recent Progress with Ethyleneoxy Phosphazenes as Lithium Battery Electrolytes. MRS Online Proc. Libr..

[11] Li Y., Chen J., Cai P., Wen Z. (2018). An Electrochemically Neutralized Energy-Assisted Low-Cost Acid-Alkaline Electrolyzer for Energy-Saving Electrolysis Hydrogen Generation. J. Mater. Chem. A.

[12] Allen C.W. (1993). The Use of Phosphazenes as Fire Resistant Materials. J. Fire Sci..

[13] Dutkiewicz M., Przybylak M., Januszewski R., Maciejewski H. (2018). Synthesis and Flame Retardant Efficacy of Hexakis(3-(Triethoxysilyl)Propyloxy)Cyclotriphosphazene/Silica Coatings for Cotton Fabrics. Polym. Degrad. Stab..

[14] Chandrasekhar V., Thilagar P., Murugesa Pandian B. (2007). Cyclophosphazene-Based Multi-Site Coordination Ligands. Coord. Chem. Rev..

[15] Allcock H.R., McIntosh M.B., Klingenberg E.H., Napierala M.E. (1998). Functionalized Polyphosphazenes:  Polymers with Pendent Tertiary Trialkylamino Groups. Macromolecules.

[16] ALOthman Z.A. (2012). A Review: Fundamental Aspects of Silicate Mesoporous Materials. Materials.

[17] Krogman N.R., Hindenlang M.D., Nair L.S., Laurencin C.T., Allcock H.R. (2008). Synthesis of Purine- and Pyrimidine-Containing Polyphosphazenes: Physical Properties and Hydrolytic Behavior. Macromolecules.

[18] Wu Z., Li L., Yan J., Zhang X. (2017). Materials Design and System Construction for Conventional and New-Concept Supercapacitors. Adv. Sci..

[19] Zhu H., Yin J., Wang X., Wang H., Yang X. (2013). Microorganism-Derived Heteroatom-Doped Carbon Materials for Oxygen Reduction and Supercapacitors. Adv. Funct. Mater..

[20] Hu L., Hou J., Ma Y., Li H., Zhai T. (2016). Multi-Heteroatom Self-Doped Porous Carbon Derived from Swim Bladders for Large Capacitance Supercapacitors. J. Mater. Chem. A.

[21] He Y., Han X., Du Y., Song B., Xu P., Zhang B. (2016). Bifunctional Nitrogen-Doped Microporous Carbon Microspheres Derived from Poly(o-Methylaniline) for Oxygen Reduction and Supercapacitors. ACS Appl. Mater. Interfaces.

[22] Zhou M., Pu F., Wang Z., Guan S. (2014). Nitrogen-Doped Porous Carbons through KOH Activation with Superior Performance in Supercapacitors. Carbon.

[23] Liu Z.-W., Peng F., Wang H.-J., Yu H., Zheng W.-X., Yang J. (2011). Phosphorus-Doped Graphite Layers with High Electrocatalytic Activity for the O_2_ Reduction in an Alkaline Medium. Angew. Chem. Int. Ed..

[24] Wu J., Yang Z., Li X., Sun Q., Jin C., Strasser P., Yang R. (2013). Phosphorus-Doped Porous Carbons as Efficient Electrocatalysts for Oxygen Reduction. J. Mater. Chem. A.

[25] Ali Z., Khan A.M., Mushtaq M.A., Wei L., Yu W.W., Wu Z. (2025). Polyphosphazene Frameworks for Sustainable Applications in Adsorption, Flame Retardancy and Electrochemistry. Nano-Struct. Nano-Objects.

[26] Yuan D., Zhou T., Zhou S., Zou W., Mo S., Xia N. (2011). Nitrogen-Enriched Carbon Nanowires from the Direct Carbonization of Polyaniline Nanowires and Its Electrochemical Properties. Electrochem. Commun..

[27] Su F., Poh C.K., Chen J.S., Xu G., Wang D., Li Q., Lin J., Lou X.W. (2011). Nitrogen-Containing Microporous Carbon Nanospheres with Improved Capacitive Properties. Energy Environ. Sci..

[28] Zhao L., Fan L.-Z., Zhou M.-Q., Guan H., Qiao S., Antonietti M., Titirici M.-M. (2010). Nitrogen-Containing Hydrothermal Carbons with Superior Performance in Supercapacitors. Adv. Mater..

[29] Qu K., Zheng Y., Jiao Y., Zhang X., Dai S., Qiao S.-Z. (2017). Polydopamine-Inspired, Dual Heteroatom-Doped Carbon Nanotubes for Highly Efficient Overall Water Splitting. Adv. Energy Mater..

[30] Li L., Yang Z., Gao H., Zhang H., Ren J., Sun X., Chen T., Kia H.G., Peng H. (2011). Vertically Aligned and Penetrated Carbon Nanotube/Polymer Composite Film and Promising Electronic Applications. Adv. Mater..

[31] Mastragostino M., Arbizzani C., Meneghello L., Paraventi R. (1996). Electronically Conducting Polymers and Activated Carbon: Electrode Materials in Supercapacitor Technology. Adv. Mater..

[32] Allcock H.R. (2006). Recent Developments in Polyphosphazene Materials Science. Curr. Opin. Solid State Mater. Sci..

[33] Li X., Lv Z., Wu M., Li X., Li Z. (2021). N, P Co-Doped Porous Carbon from Cross-Linking Cyclophosphazene for High-Performance Supercapacitors. J. Electroanal. Chem..

[34] Liu W., Zhang S., Dar S.U., Zhao Y., Akram R., Zhang X., Jin S., Wu Z., Wu D. (2018). Polyphosphazene-Derived Heteroatoms-Doped Carbon Materials for Supercapacitor Electrodes. Carbon.

[35] Bagotsky V.S., Skundin A.M., Volfkovich Y.M. (2015). Electrochemical Power Sources: Batteries, Fuel Cells, and Supercapacitors.

[36] Karpova E.A., Sysoev A.A., Rozhkov I.M., Soldatov M.A. (2025). Preparation of Three-Dimensional Porous Amino-Substituted Cyclophosphazenes. INEOS OPEN.

[37] Hakimi M., Rezaei H., Moeini K., Mardani Z., Carpenter-Warren C. (2020). Solvent Free Synthesis of Three Cyclotriphosphazene Derivatives Containing Piperazine Substituents Using Microwave Irradiation. Spectral, Theoretical, Solution and Docking Studies. Phosphorus Sulfur Silicon Relat. Elem..

[38] Mehmood S., Wang L., Yu H., Haq F., Amin B., Uddin M.A., Fahad S., Haroon M., Shen D., Ni Z. (2022). Preparation of Poly(Cyclotriphosphazene-Co-Piperazine) Nanospheres and Their Drug Release Behavior. Int. J. Polym. Mater. Polym. Biomater..

[39] Yang R., Ma B., Zhang X., Li J. (2019). Fire Retardance and Smoke Suppression of Polypropylene with a Macromolecular Intumescent Flame Retardant Containing Caged Bicyclic Phosphate and Piperazine. J. Appl. Polym. Sci..

[40] Wu F., Zhao Y., Hou Z., Jiang M., He W., Su D., Wang M., Wang J.-G. (2025). Lignocellulosic Oxidation Bridging to Modulate Pseudographitic Domain of Hard Carbon toward Boosted Sodium Storage. J. Energy Storage.

[41] Barsukov I.V., Johnson C.S., Doninger J.E., Barsukov V.Z., Barsukov I.V., Johnson C.S., Doninger J.E., Barsukov V.Z. (2006). New Carbon Based Materials for Electrochemical Energy Storage Systems: Batteries, Supercapacitors and Fuel Cells.

[42] Hussain C.M., Ahamed M.B., Hussain C.M., Ahamed M.B. (2024). Functionalized Nanomaterials Based Supercapacitor: Design, Performance and Industrial Applications.

[43] Shirley D.A. (1972). High-resolution X-ray photoemission spectrum of the valence bands of gold. Phys. Rev. B.

[44] Scotfield H. (1976). Hartree-Slater subshell photoionization cross-sections at 1254 and 1487 eV. J. Electr. Spectr. Relat. Phenom..

