# Supplementary material for: Phosphazene-Based Porous Polymer as Electrode Material for Electrochemical Applications
Source: Polymers (Basel). 2026 Jan 29;18(3):366. doi: 10.3390/polym18030366 (PMC12899548; doi:10.3390/polym18030366)
Supplement: Supplementary file 1 [file polymers-18-00366-s001.zip › polymers-4050820-supplementary.pdf]

Supporting Information

The Use of Phosphazene-Based Porous Polymer in Electrode Materials for Electrochemical Devices

Ekaterina A. Karpova [1], Alexander A. Sysoev [1], Ilya D. Tsvetkov [1], Alexey L. Klyuev [1,2], Oleg A. Raitman [1,2] and Mikhail A. Soldatov [1]*

[1] Mendeleev University of Chemical Technology of Russia
[2] A. N. Frumkin Institute of Physical Chemistry and Electrochemistry of the Russian Academy of Sciences
* Correspondence: soldat89.89@gmail.com

Characterization methods.

FT-IR spectra were obtained on a Jasco FT-IR-4600 infrared spectrophotometer with a wavelength range of 4600 to 300 cm-1.

X-ray diffraction data were obtained using Equinox 100 diffractometer. The source was a copper target x-ray tube, monochromatized to the Cu Ka (1.54060 Å). Imaging of powder samples, as well as the determination of the size and shape of their particles, was carried out on a scanning electron microscope (Thermo Fisher Scientific Quattro C) at an accelerating voltage of 30 kV, a probe current of 0.98 nA in high vacuum, with pre-spraying of a 10 nm thick layer of carbon. The carbon layer was sputtered on a compact sputtering machine, Safematic CCU-010 LV. To determine the elemental composition of the powder particles, energy dispersive X-ray microanalysis (EDX) was performed in high vacuum mode using an analytical X-ray detector, UltraDry, from Thermo Scientific.

N_2 sorption isotherm measurements were characterized with a Anton Paar Nova 600 BET surface area and pore-size analyzer. Before measurements, the samples were degassed for 6 h at 150 °C under vacuum. A sample of ca.100 mg and UHP-grade N2 (99.999%) gas source were adopted in the N_2 sorption measurements at 78.15 K. BET surface areas were confirmed over a P/P_0 range from 0 to 1.

XPS data were obtained using OMICRON ESCA+ spectrometer (Germany) with Al anode and equipped with monochromatic X-rat source AlKα XM1000 (emission energy 1486.6 eV and power 252 W). Registration of spectra was carried out with semispherical detector Argus. Transmittance energy of detector was 50 eV for wide spectrum and 20 eV for other spectra. The pressure in the analyzer chamber did not exceed 10^{-9} mbar. Background subtraction was performed by the Shirley method [43]. For quantitative measurements Scotfield coefficients were used [44].

To study and control the state of the surface of the material at the electrode/electrolyte interface, cyclic voltammograms (CVA) were measured on a P-45X potentiostat/galvanostat (“Electrochemical instruments”, Chernogolovka, Russia), at a potential sweep rate of 50 mV/s, in 0.5 M H_2SO_4, at room temperature

(thermostated cell, 25 ^{0}C) in an argon atmosphere, on the standing RDE. The standard three-electrode hermetic cell for RDE was used. The 4 cm^2 Pt mesh was used as counter electrode and a saturated Ag/AgCl electrode as reference, relative to which the potentials are given. All voltages were recalculated vs. reversible hydrogen electrode (**RHE**). The electrode specific capacity can be obtained from the double-layer region on CVA (the current curve is parallel to the potential axis), then current value is normalized to the potential scan rate and to the mass of the carbon material, taking into account the background current of the substrate (blank RDE). The charging current values averaged in the 0.8 – 1.0 V potential range (in the sweep section from the cathode potential region to the anode region) are taken from the CVA obtained in an oxygen-free solution. The charging current values previously obtained under similar conditions for a blank RDE without the applied carbon material were subtracted from the sample current values obtained. The adjusted values were further recalculated into the specific capacity C of the carbon material using the formula

$$C = \frac{I}{\upsilon \times m} \qquad \text{(S1)}$$

where C is the specific capacity, F/g; I is the adjusted value of the charging current, A; $\upsilon = 0.05$ V/s is the potential scan rate; $m = 1.26\times10^{-5}$ g is the mass of the carbon sample on the disk electrode.

To study the electrocatalytic properties in the oxygen reduction reaction **(OER)**, the working solution was saturated (purged) with oxygen and polarization curves were taken, under similar conditions, but under rotation of the disk electrode. A significant parameter is the value of either the limiting current or the current at the minimum potential. The magnitude of the current is the rate of the electrochemical reaction: the higher it is, the more active the electrocatalyst is. After CVA data collection, the solution was oxygenated for 20 minutes, the disk electrode was rotated (1500 rpm), and the polarization curves of oxygen reduction from the open circuit potential **(OCP)** to the potential E = 0.07 V. The electrocatalytic activity of

the carbon material was determined as the final value of the current of the polarization curve, related to the mass of the carbon material sample:

$$A = \frac{I_{ox}}{m} \tag{S2}$$

where A is the electrocatalytic activity, A/g: I_{ox} is the final value of the current of the polarization curve; m = 1.26×10^{-5} g is the mass of the carbon sample on the RDE surface. The dimension is A/g. The background current of blank RDE is negligible.

[43] D.A. Shirley, High-resolution X-ray photoemission spectrum of the valence bands of gold. Phys. Rev. B., 1972, 5, 4709-4713. https://doi.org/10.1103/PhysRevB.5.4709

[44] H. Scotfield, Hartree-Slater subshell photoionization cross-sections at 1254 and 1487 eV. J. Electr. Spectr. Relat. Phenom., 1976, 8, 129-137 https://doi.org/10.1016/0368-2048(76)80015-1

Table S1. Element content on surface of PIP-C obtained from XPS data

Peak name		Eb, eV	Quant., at.%	
C1s	C=C	284.2	51.4	70.3
	C-C	285.0	4.6	
	C-N	285.7	3.0	
	C-O	286.0	5.5	
	C=O	287.2	3.4	
	C(O)O	288.2	1.5	
	C(O)OH	289.2	0.9	
N1s	P=N-P	398.3	2.8	7.5
	P-N(H)-C	399.8	1.7	
	P-NH2	401.3	2.3	
	R-NO2	404.6	0.7	
O1s	C=O	530.5	5.9	15.2
	C-O	532.1	7.9	
	R-NO2	532.6	1.3	
P2p	phosphazene	132.8	7.0	7.0

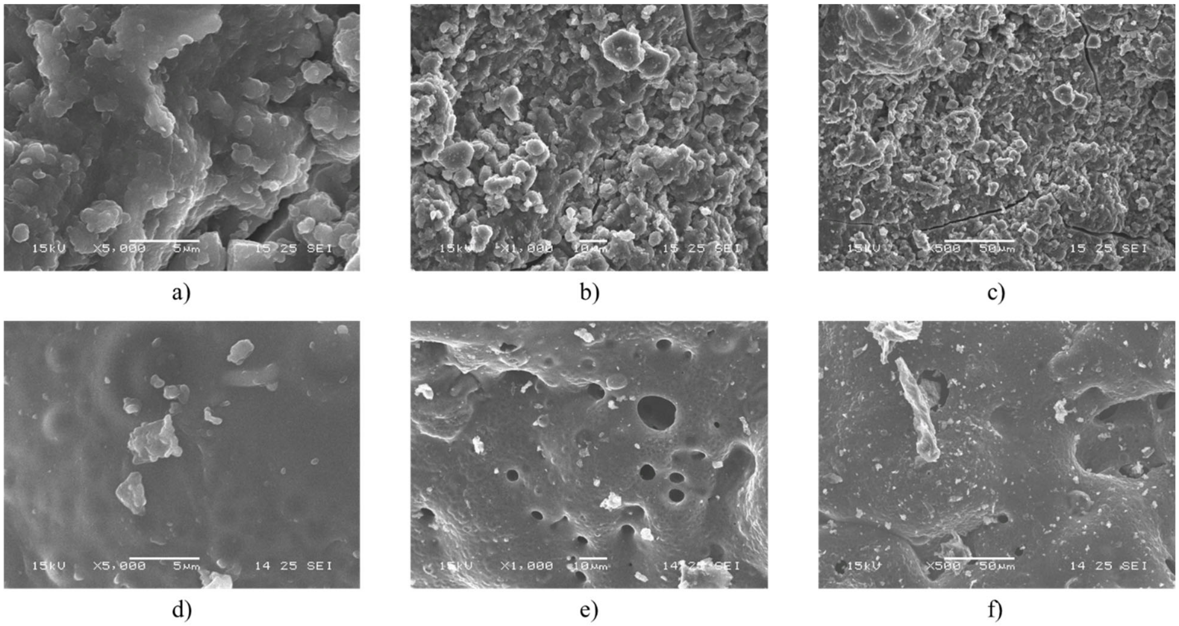

Figure S1. SEM images of PIP (a, b, c) and PIP-C (d, e, f)

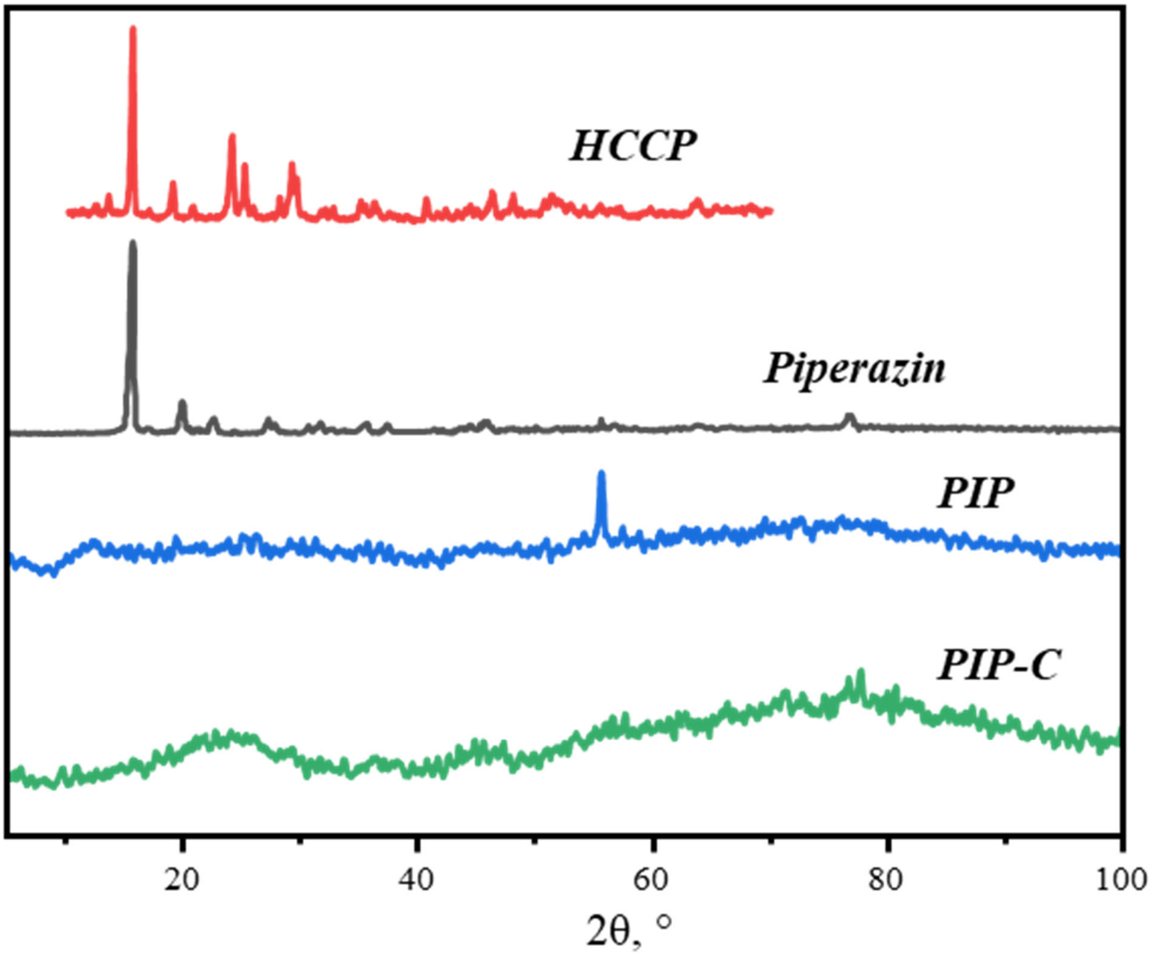

Figure S2 XRD spectra of initial monomers and obtained products

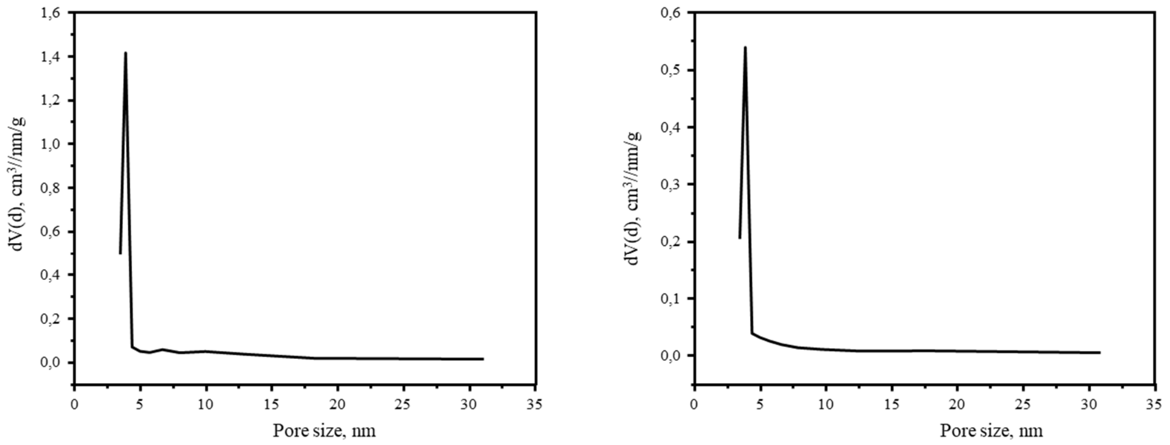

a) b)

Figure S3. Pore size distributions for PIP (a) and PIP-C (b) calculated by using BJH method

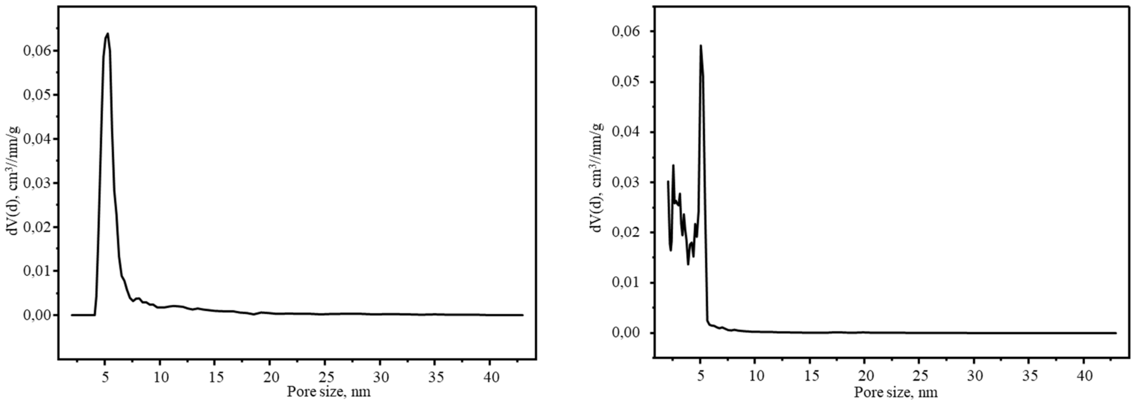

a) b)

Figure S4. Pore size distributions for PIP (a) and PIP-C (b) calculated by using DFT method with the use of the model of cylindrical geometry of pores
